# Bushen-Tiansui Formula Improves Cognitive Functions in an A*β*_1–42_ Fibril-Infused Rat Model of Alzheimer's Disease

**DOI:** 10.1155/2020/8874885

**Published:** 2020-09-24

**Authors:** Chenxia Sheng, Panpan Xu, Xinyi Liu, Weijun Peng, Daxiong Xiang, Shilin Luo

**Affiliations:** ^1^Department of Integrated Traditional Chinese and Western Medicine, The Second Xiangya Hospital, Central South University, Changsha 410011, China; ^2^Department of Pharmacy, The Second Xiangya Hospital, Central South University, Changsha 410011, China; ^3^Hunan Provincial Engineering Research Center of Translational Medicine and Innovative Drug, Changsha 410011, China

## Abstract

Bushen-Tiansui Formula (BTF) was empirically updated from a classical prescription named Kong-Sheng-Zhen-Zhong pill. It is based on the traditional Chinese medicine theory of the mutual relationship between the brain and the kidney and is intended to treat neurodegenerative diseases. This formulation has been used for several years to treat patients with Alzheimer's disease- (AD-) like symptoms in our clinical department. However, the medicinal ingredients and the mechanisms by which BTF improves cognition and memory functions have not been characterized. In this study, we used UPLC-MS to generate a chromatographic fingerprinting of BTF and identified five possible active ingredients, including stilbene glycoside; epimedin A1, B, and C; and icariin. We also showed that oral administration of BTF reversed the cognitive defects in an A*β*_1–42_ fibril-infused rat model of AD, protected synaptic ultrastructure in the CA1 region, and restored the expression of BDNF, synaptotagmin (Syt), and PSD95. These effects likely occurred through the BDNF-activated receptor tyrosine kinase B (TrkB)/Akt/CREB signaling pathway. Furthermore, BTF exhibited no short-term or chronic toxicity in rats. Together, these results provided a scientific support for the clinical use of BTF to improve learning and memory in patients with AD.

## 1. Introduction

Alzheimer's disease (AD) is the leading cause of dementia worldwide, but the etiology and pathogenesis of this disease have not been characterized. Accumulation of *β*-amyloid peptide (A*β*) in the brain and hyperphosphorylation and cleavage of the microtubule-associated protein Tau are hallmarks of AD [1]. However, over the last decade, a series of new drugs designed to clear neurofibrillary tangles have failed to improve or reverse AD, which indicated that neurofibrillary tangles correlated weakly with the degree of dementia in patients with AD [2]. In contrast, synaptic loss has been strongly correlated with cognitive impairment, and it may be the pathological basis of cognitive changes in AD [3].

Neurotrophins are growth factors that regulate neuronal development, differentiation, and survival. Brain-derived neurotrophic factor (BDNF) is an important neurotrophin that is distributed extensively throughout the central nervous system. BDNF binds to receptor tyrosine kinase B (TrkB) and triggers activation of the downstream TrkB/Akt and TrkB/CREB signaling pathways, resulting in the synthesis of synaptotagmin (Syt) and PSD95 in synapses. Synaptotagmin and PSD95 confer protection by regulating the repair of synapses and the reconstruction of neural circuits to improve learning and memory in animals with dementia [4–6]. Studies have shown that the expression of BDNF was reduced in the brains of patients with AD with synaptic loss [7]. A strategy of using BDNF as a therapeutic agent for neurologic disorders was carried out based on this preclinical evidence. Unfortunately, the outcomes of several clinical trials involved the intrathecal infusion of recombinant BDNF to treat patients with amyotrophic lateral sclerosis have been disappointing due to the short *in vivo* half-life and poor delivery of BDNF [8, 9]. Thus, novel strategies to directly stimulate production and expression of BDNF by exploring drugs may result in better therapeutic outcomes.

Facing the treatment complexity of AD, a growing body of reports have suggested that traditional Chinese medical formulas (TCMFs) with multitarget effects may result in improved cognitive function [10–12]. Bushen-Tiansui Formula (BTF) is derived from a classic prescription, Kong-Sheng-Zhen-Zhong pill (Qianjin Formulas), and was empirically modified from this classic prescription by adjusting the composition and proportion of herbs. Its formula is intended to meet neurodegenerative diseases and embodies the Chinese medicine theory of the mutual relationships between the brain and the kidney, in which the core statements are that the deficiency of kidney function leads to the encephalon loss, and the kidney gives birth to the encephalon and the brain stores marrow [13]. BTF has been utilized for several years to treat patients with AD-like symptoms in our clinical department. Nonetheless, the pharmacological ingredients in BTF, and the mechanisms by which they improve cognitive and memory functions, have not been characterized. Our previous study reported that icariin, a major active component from *Herba Epimedium brevicornum* (Yin-Yang-Kuo) that belongs one of the herbs in BTF, improved synaptic plasticity in an A*β*_1–42_ rat model of AD [14]. As a single compound, however, it will be a long-term task to develop it into an innovative promising drug for clinical use.

In this present study, we characterized the formula composition of BTF and generated a chromatographic fingerprint profile. We also evaluated the effects of BTF on cognition and memory functions in a rat model of AD and evaluated BFT-induced expression of BDNF and the activation of the TrkB/Akt/CREB cascaded signaling pathways. Our study provided scientific support for the clinical use of BTF to improve learning and memory in patients with AD.

## 2. Materials and Methods

### 2.1. Preparation of BTF Extract

**Figure 1:**
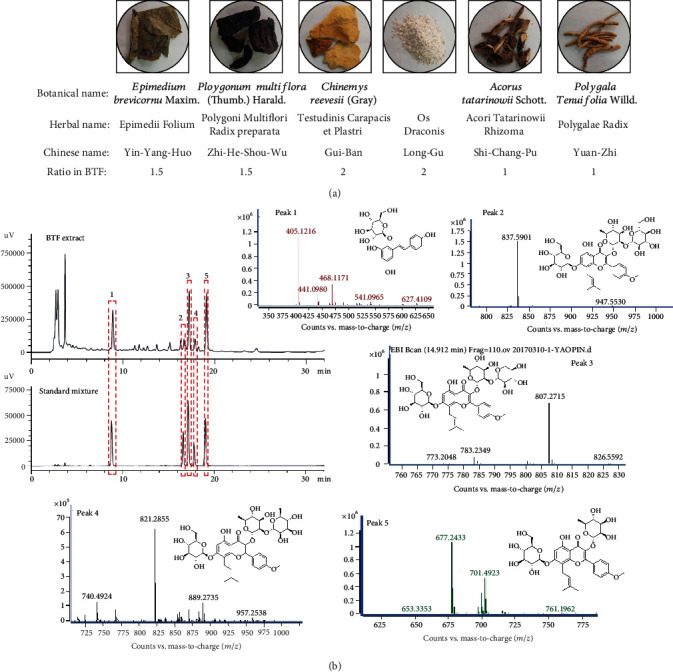
Herb information and chromatographic fingerprinting of the Bushen-Tiansui Formula (BTF). (a) Representative figures, herbal names, and Chinese names and the ratio of the corresponding herbs in BTF. (b) Chromatograms of BTF and a standard mixture, as well as *m*/*z* ratio and structure information of peaks 1-5 in the BTF chromatogram. Peak 1: stilbene glycoside; peak 2: epimedin A1; peak 3: epimedin B; peak 4: epimedin C; peak 5: icariin.

BTF is comprised of six herbs mixed in the proportions summarized in Figure 1(a). All the mentioned botanical names can be checked following the database of http://www.theplantlist.org. The herbal names and Chinese names were retrieved from the 2015 edition of the Chinese pharmacopeia. All herbs in BTF were purchased from the TCM pharmacy of the Second Xiangya Hospital, Central South University (CSU), Changsha, Hunan Province. Voucher specimens (201605301-6) were well deposited at the department of integrated traditional Chinese and Western medicine at the Second Xiangya Hospital, CSU. The herbs were soaked in a 10 times volume of ddH_2_O (*w*/*v*) for 1 h and then boiled twice for 1 h each. The two boiled solutions were combined and concentrated under vacuum and then freeze-dried to yield a lyophilized powder (output rate of 14.3% (*w*/*w*)). The lyophilized powder of BTF was stored at -20°C until used.

### 2.2. Chromatographic Fingerprint Analysis of BTF Extract

Ultrahigh-performance liquid chromatography-tandem mass spectrometer (UPLC-MS) was utilized to analyze the principal components in BTF. Standard compounds including stilbene glycoside (BWB50367), epimedin A1 (BWB50192), epimedin B (ASB-5159-010), epimedin C (ASB-5160-010), and icariin (GBW09541) were purchased from the National Standard Center. A CNW Athena C_18_-WP column (4.6 mm × 150 mm, 5 *μ*m) was used as a solid phase and maintained at 35°C while the spectrum analysis was performed. The mixture of water (A) and CH_3_CN (B) was acted as a mobile phase with a gradient elution ratio as follows: 0-10 min 20%-30% B, 10-22 min 30% B, 22-25 min 30-33% B, and 25-30 min 33-80% B. The monitoring wavelength was 270 nm with a flow rate of 0.5 mL/min. Electrospray ionization (ESI) mode was used for mass spectrometry with a capillary voltage of 3500 V.

### 2.3. A*β*_1-42_ Preformed Fibril Preparation

A*β*_1–42_ (Sigma, USA) was dissolved at 1 mg/mL in hexafluoroisopropanol (HFIP) at room temperature and then sonicated in a bath sonicator for 5 min. The HFIP was evaporated using a gentle stream of nitrogen gas, and then nine volumes of ice-cold distilled water were added while vortexed occasionally. Keeping the solution on ice for 30 min, one volume of 10x fibril-forming buffer (0.2 M NaPi, 1.5 M NaCl, 0.2% NaN_3_, pH 7.5) was added and vortexed repetitively. We sealed the solution tube and stored at 37°C for one week and vortexed daily. Fibril formation was verified using a Thioflavin T binding assay according to a previous report [15]. The A*β* fibrils were stored at -80°C.

### 2.4. A*β*_1–42_ Fibril-Infused Rat Model and BTF Treatment

**Figure 2:**
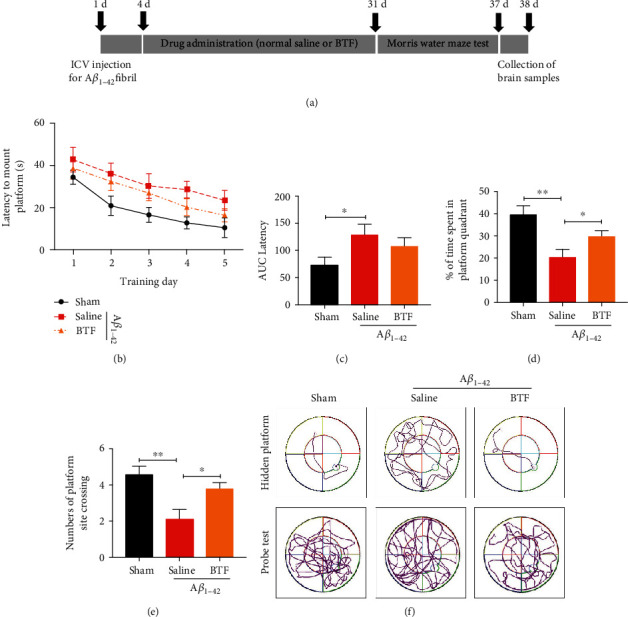
Oral administration of BTF rescues cognitive deficits in A*β*_1–42_ fibril-infused rats. (a) The schedule of the treatment schedule and behavioral evaluation. (B-E) BTF improved cognitive functions in A*β*_1–42_ fibril-infused rats. Rats (*n* = 8 or 9 per group) orally administered saline or BTF were trained in the water maze for 5 d. Data are presented as means ± SEM of latency to mount the escape platform (b), the AUC of latency (c), the percentage of time spent (d) (*F*(2, 20) = 5.493, *p* = 0.0126) and the number of crosses in the target quadrant (e) (*F*(2, 20) = 7.216, *p* = 0.0044). (f) Representative images of the swim paths of different rat groups in the hidden platform and probe tests. ^∗^*p* < 0.05, ^∗∗^*p* < 0.01 by one-way ANOVA with Tukey's multicomparison test compared with saline-treated A*β*_1–42_ fibril-infused rats.

The A*β*_1–42_ fibril-infused rat model was established as described in our previous study [14]. In brief, adult male Sprague-Dawley (SD) rats (weight: 200−220 g) were anesthetized with isoflurane and then fixed in a stereotactic apparatus. The A*β*_1–42_ fibrils (3 *μ*L) were delivered bilaterally into the lateral ventricles at a rate of 0.5 *μ*L/min (from bregma, anteroposterior (AP) −1.0 mm, 1.5 mm lateral to the sagittal suture, and 4.6 mm beneath the dura). An equivalent volume of sterile saline was injected as a sham group (*n* = 8). Following infusion for 3 days, rats that received the A*β*_1–42_ infusion were randomly distributed to two groups (*n* = 8 for each group). According to clinical use dose, the test group was orally administered 27 g/kg BTF, and another group was orally administered an equivalent volume of sterile saline. The animals were dosed once per day for 28 days. The rats were assigned to gender- and age-matched treatment groups using a randomized block design. The total experimental period is summarized in Figure 2(a), and the experimental procedures were approved by the Review Committee of Central South University (Changsha, China).

### 2.5. Morris Water Maze Test

The Morris water maze test was carried out from 28 days after the beginning of BTF intervention. Rats in three groups were trained in a round, diluted ink water-filled tub (160 cm diameter × 90 cm height) in an environment rich with extra maze cues. An invisible escape platform (12 cm diameter × 25 cm height) was located in a fixed spatial location 1 cm below the water surface independent to utilize extra maze cues to determine the location of the platform. At the beginning of each trial, the rats were placed in the water maze with their paws touching the wall from one of four different starting positions (N, S, E, and W). Each rat was subjected to four trials per day for five consecutive days with a 15-minute intertrial interval. The maximum trial length was 60 s, and the rats were manually guided to if they did not reach the platform in the allotted time. Upon reaching the invisible escape platform, the rats were left on it for an additional 10 s to allow for a survey of spatial cues in the environment to guide future navigation to the platform. The temperature of the water was monitored every hour and maintained between 22 and 25°C. Following the 5 days of task acquisition period, a probe trial was presented when the platform was removed, and the number of platform crossings and the percentage of time spent in the quadrant that previously contained the escape platform during task acquisition was recorded over 90 s. The whole trial process and the analysis of behavioral parameters were recorded through the ANY-maze video tracking system (Stoelting Co., USA).

### 2.6. Electron Microscopy

Synaptic ultrastructure detection was determined by electron microscopy as described previously [16]. Briefly, after deep anesthesia, rats were perfused transcardially with 4% paraformaldehyde in PBS. Hippocampal slices were postfixed in cold 2.5% glutaraldehyde, then dehydrated, soaked, and embedded through a graded acetone series. The embedded sections were dual-stained with uranyl acetate and lead citrate and visualized at 100 kV in a transmission electron microscope (Hitachi Ltd., Tokyo, Japan). Synapses were evaluated by the presence of synaptic vesicles and postsynaptic density, including the number of synapses, the width of each synaptic cleft, the thickness of the postsynaptic density, and the length of the synaptic active zone.

### 2.7. Western Blotting

Western blotting was performed using a standard protocol. Rat hippocampus tissue was sonicated and lysed with RIPA lysis buffer, and insoluble pellets were removed by centrifugation at 15,000 × g for 15 min at 4°C. Protein concentration was measured using the BCA method, and the lysates were stored at -80°C until analysis. Equal amounts of protein (30-40 *μ*g) were loaded for blotting with anti-p-TrkB/TrkB (1 : 1000, #sc-8058/#sc-7268, Santa Cruz Biotechnology, CA, USA), anti-p-Akt/Akt (1 : 1000, #4060/#9272), anti-p-CREB/CREB (1 : 500, #9189/#9197), anti-Syt (1 : 1000, #14558), and anti-PSD-95 (1 : 1000, #2507) (Cell Signaling Technology, Denver, MA, USA), and anti-BDNF (1 : 500, #108319, Abcam, Cambridge, UK).

### 2.8. Immunohistochemistry

Immunohistochemistry (IHC) was performed to visualize BDNF and p-Akt according to the manufacturer's instructions (Invitrogen). Briefly, free-floating 25 *μ*m thick serial hippocampus sections were treated with 0.3% hydrogen peroxide for 10 min, and then, sections were rinsed three times with PBS and blocked in Reagent 1A for 10 min followed by incubation with BDNF (1 : 300) or p-Akt (1 : 500) antibody at 4°C overnight. After PBS washing, sections incubated with a biotinylated second antibody Reagent 1b followed by the conjugate enzyme Reagent 2 for each 10 min. Finally, a chromogen AEC single solution was utilized to develop the signals and captured in a microscope (BX51TF, Olympus, Tokyo, Japan) with cellSens standard V3 detection system.

### 2.9. Hematoxylin and Eosin Staining

Multiple organs were collected and were immediately fixed in 4% formaldehyde. After immersion, organs were dehydrated by gradual soaking in alcohol and xylene, embedded in paraffin, and then sliced into 5 *μ*m thick sections, which were stained with standard hematoxylin and eosin (H&E) staining protocol [17]. Sections were visualized under a digital optical microscope (Olympus, Tokyo, Japan).

### 2.10. Statistical Analysis

Statistical analysis was performed with Prism 7.0 (GraphPad software). All data were expressed as means ± SEM. from three or more independent experiments. Histological data were analyzed by one-way ANOVA. The threshold for significance for all experiments was set at ^∗^*p* < 0.05, and smaller *p* values were represented as ^∗∗^*p* < 0.01 and ^#^*p* < 0.001.

## 3. Results

### 3.1. Chromatographic Fingerprinting Analysis of BTF

The pharmacological effect of traditional Chinese medicine formulas (TCMFs) is derived from combinations of active compounds. To investigate the possible major medicinal compounds in BTF, a qualitative assessment of ingredients was tentatively characterized the by UPLC-MS system and the chromatographic fingerprint was established as illustrated in Figure 1(b). Approximately 14 chromatographic peaks can be defined in the characteristic profile of BTF. According to the *m*/*z* ratio in the MS detection, five of these peaks (peak 1-5) were identified as stilbene glycoside (peak 1, *m*/*z* 405.1216 [M-H]^−^), epimedin A1 (peak 2, *m*/*z* 837.5901 [M-H]^−^), epimedin B (peak 3, *m*/*z* 807.2715 [M-H]^−^), epimedin C (peak 4, *m*/*z* 821.2855 [M-H]^−^), and icariin (peak 5, *m*/*z* 677.2433 [M+H]^+^) (Figure 1(b)). These compounds were chemical components of *Radix Polygoni Multiflori* Preparata and *Herba Epimedii Brevicornus* based on the previous phytochemistry studies [18, 19]. Further, standard substances for these five compounds were purchased and their mixed solution was subjected to UPLC-MS analysis with the same elution conditions. The comparison of chromatograms showed similar UV absorption spectra and retention times for each peak. Therefore, these data suggested that stilbene glycoside; epimedin A1, B, and C; and icariin were characteristic components in BTF.

### 3.2. Oral Administration of BTF Rescues Cognitive Deficits in A*β*_1–42_ Fibril-Infused Rats

To evaluate the effects of BTF on cognitive function in the AD model, hippocampus-dependent spatial memory in A*β*_1–42_ fibril-infused rats was assessed using the Morris water maze test. The average escape latency to the hidden platform for each of the five acquisition days was calculated and plotted (Figures 2(b) and 2(c)). Two-way mixed ANOVA (group × training day) for latency revealed a main effect of the training day (*p* < 0.05) and of the group (*p* < 0.05), but there was no interaction (Figure 2(b)). The AUC of the escape latency was significantly greater in the saline-treated A*β*_1–42_ fibril-infused rats compared with that in the sham group, which indicated impaired acquisition of the spatial learning following intracerebroventricular injection of A*β*_1–42_ fibrils. Memory recall for the platform location was assessed in the probe trial by removing the platform and allowing the rats to search for 90 s. Compared with the sham group, saline-treated A*β*_1–42_ fibril-infused rats spent a significantly lower percentage of their time and fewer platform site crossing in the quadrant that formerly contained the hidden platform, which was indicative of severe deficits in spatial memory recall. Compared with the sham group rats, the A*β*_1–42_ fibril-infused rats treated with BTF spent a significantly greater percentage of time in the target quadrant and crossed the target quadrant more frequently (Figures 2(d)–2(f)), which demonstrated the rescue of spatial memory.

### 3.3. Oral Administration of BTF Prevents Synaptic Loss in A*β*_1–42_ Fibril-Infused Rats

**Figure 3:**
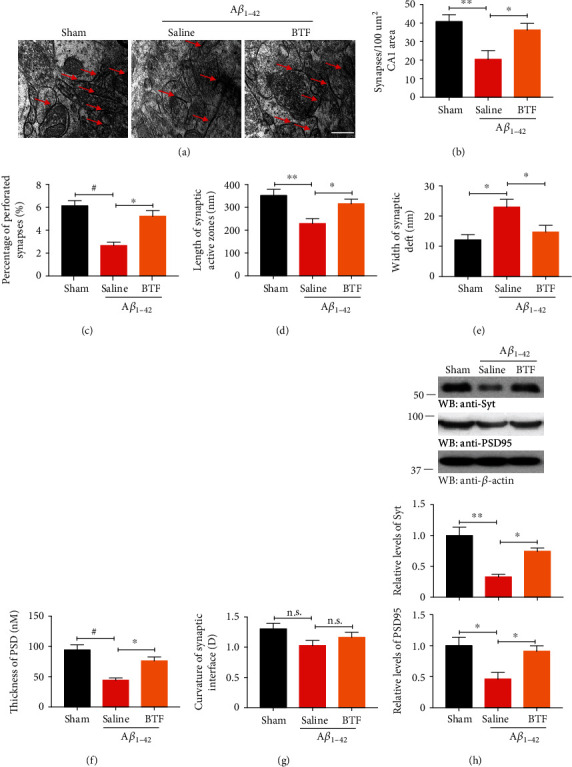
Oral administration of BTF prevents synaptic loss in A*β*_1–42_ fibril-infused rats. (a) Representative electron micrographs of synapses. Red arrows indicate the synapses. Scale bar, 1 *μ*m. (b–g) Quantitative analysis of the synaptic density (b) (*F*(2, 12) = 7.1, *p* = 0.0092) and the parameters of synaptic structure in the AC1 region. Data are presented as means ± SEM for the percentage of perforated synapses (c) (*F*(2, 12) = 18.27, *p* = 0.0002), synaptic active zone length (d) (*F*(2, 12) = 7.689, *p* = 0.0071), synaptic cleft width (e) (*F*(2, 12) = 6.732, *p* = 0.0110), PSD thickness (f) (*F*(2, 12) = 15.24, *p* = 0.0005), and synaptic interface curvature (g). *n* = 5 in each group. (h) Immunoblotting analysis of synaptic markers in brain homogenates from rats treated with saline or BTF. BTF treatment increased the expression of synaptic markers in A*β*_1–42_ fibril-infused rats. ^∗^*p* < 0.05, ^∗∗^*p* < 0.01 and ^#^*p* < 0.001 by one-way ANOVA with Tukey's multicomparison test compared with saline-treated A*β*_1–42_ fibril-infused rats. Western blot data are representative of three independent experiments.

Synaptic loss and decreased hippocampal synaptic plasticity are believed to be the basis of cognitive impairment in the early phases of AD [20]. We directly quantified the synaptic density and evaluatedsynaptic ultrastructure parameters in the CA1 region of the hippocampus using electron microscopy (EM). Saline-treated A*β*_1–42_ fibril-infused rats showed significantly reduced synaptic density, perforated synapses, synaptic active zones length, and postsynaptic density thickness and increased synaptic cleft width. The oral administration of BTF significantly reversed these ultrastructure changes but did not alter the curvature of the synaptic interface (Figures 3(c)–3(g)). To confirm these findings, we performed immunoblotting for the presynaptic marker synaptotagmin and the postsynaptic marker PSD95. Saline-treated A*β*_1–42_ fibril-infused rats displayed the considerably decreased expression of synaptotagmin and PSD95, which was indicative of synaptic degeneration in this AD model. Treatment with BTF reversed A*β*_1–42_ fibril-induced reduction of synaptic marker expression (Figure 3(h)). These results indicated that oral administration of BTF inhibited synaptic loss and improved synaptic plasticity in A*β*_1–42_ fibril-infused rats.

### 3.4. BTF Promotes BDNF Expression and Activates Downstream Signaling Pathways in the Rat Brain

**Figure 4:**
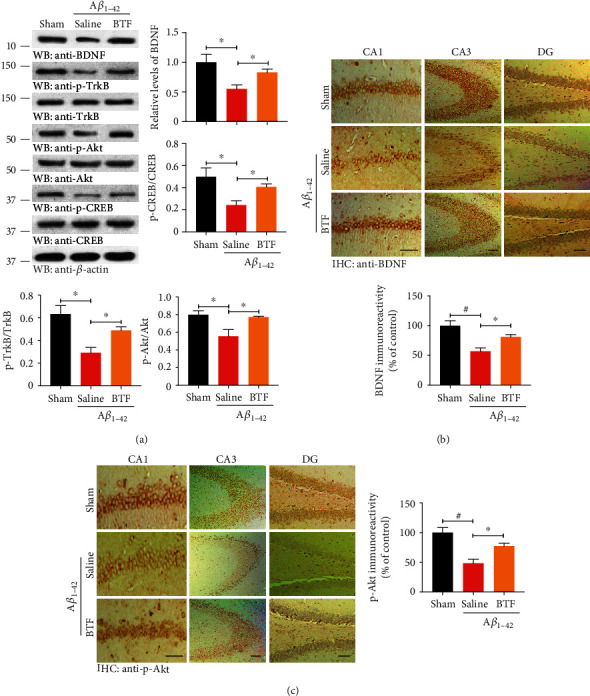
BTF promotes BDNF expression and activates downstream signaling pathways in the rat brain. (a) BDNF-TrkB signaling pathways were analyzed by immunoblotting with the indicated antibodies. The expression of BDNF induced by BTF triggered the phosphorylation of TrkB, Akt, and CREB. (b, c) IHC staining for BDNF (b) and p-Akt (c) in different hippocampal regions. BTF treatment significantly escalated their expression in A*β*_1–42_ fibril-infused rats, which were consistent with Western blot results. Quantification of BDNF intensity (*F*(2, 12) = 12.21, *p* = 0.0013) and p-Akt intensity (*F*(2, 12) = 14.27, *p* = 0.0007) are shown. Data are represented as of 12-18 sections prepared from three rats in each group. Scale bar, 150 *μ*m. ^∗^*p* < 0.05, ^#^*p* < 0.001 by one-way ANOVA with Tukey's multicomparison test. Western blot data are representative of three independent experiments.

To explore the possible mechanisms by which BTF improved cognitive function in A*β*_1–42_ fibril-infused rats, we investigated BDNF and its downstream signaling pathways. Following behavioral testing, we monitored BDNF expression in rat brains by immunoblotting analysis using an anti-BDNF antibody. Surprisingly, quantitative analysis revealed that BDNF was regained to normal levels in BTF-treated A*β*_1–42_ fibril-infused rats. Furthermore, the expression of phosphorylated TrkB, but not total TrkB, was notably elevated following BTF treatment (Figure 4(a), 1-3^th^ panels). As expected, the main proteins on TrkB signaling pathway were more prominently phosphorylated in A*β*_1–42_ fibril-infused rats treated with BTF than in those treated with saline, as were the downstream activation of Akt/CREB signaling cascades (Figure 4(a)), which resulted in the synthesis of PSD95 in synapses. These results were confirmed by immunohistochemistry (IHC) staining of rat hippocampi using anti-BDNF (Figure 4(b)) and p-Akt S473 (Figure 4(c)). Therefore, these data indicated that the promotion of BDNF expression by BTF treatment led to the activation of its downstream TrkB-Akt/CREB signaling cascades might be responsible for the cognitive function improvement in AD rats.

### 3.5. Oral Administration of BTF Presents No Toxicity for Rats

**Figure 5:**
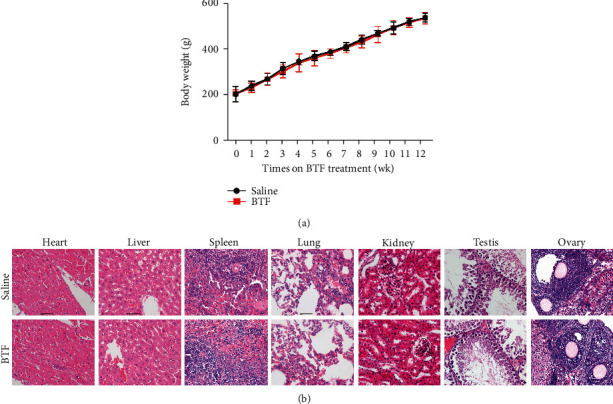
Oral administration of BTF presents no toxicity for rats. (a) The monitoring curve of body weight demonstrated no significant difference between saline and BTF treatment groups. (b) Representative images of H&E staining of tissue sections from multiple organs. SD rats (200–220 g) were treated with a daily dose of 54 g/kg of BTF for 12 consecutive weeks (*n* = 6). Continuous weekly weight records were kept, and the heart, liver, spleen, lung, kidney, testis, and ovary were collected for pathological analysis at the end of the study.

Drug safety is an important consideration in clinical investigations. As a compulsory experiment, we performed a 12-week chronic BTF toxicity study in Sprague-Dawley (SD) rats (200–220 g) with a daily dose of 54 g/kg. Continuous weekly weight records and the H&E staining of tissue sections from multiple organs (the heart, liver, spleen, lung, kidney, testis, and ovary) showed that there were no significant differences between rats administered with BTF and those administered with saline (Figures 5(a) and 5(b)). Besides, blood levels of RBC, HB, WBC, and ESR were within the normal ranges in rats that received BTF (data not shown). Thus, this study supports that the oral administration of BTF is safe and trustworthy for treating AD.

## 4. Discussion

Traditional Chinese medical formulas (TCMFs), developed based on the theory of the holistic body in traditional Chinese medicine (TCM), have been historically proven to be effective drugs in treating human diseases. Kidney-brain communication and reciprocity is one of the most important theories in TCM. This theory states that the kidney is a producer of the encephalon, and the cerebral marrow will be sufficient if the spirit in the kidney is exuberant [13]. Modern medical epidemiological data has shown that individuals at all stages of chronic kidney disease (CKD) are at higher risk of developing cognitive disorders and dementia [21]. Studies have proven that vascular injury, endothelial dysfunction, and direct neuronal toxicity may be potential factors in the pathophysiologic link of the kidney-brain axis [22–24]. “Kong-Sheng-Zhen-Zhong” pill was detailed in the classical medical book “Qianjin Formulas” written by Sun Simiao, a famous ancient Chinese medical expert. This formula is comprised of many nourishing herbs to achieve the treatment objectives that replenish vital essence, tonify kidney yin, and nourish the bone marrow [25]. Bushen-Tiansui Formula is an empirically improved version of the Kong-Sheng-Zhen-Zhon pill developed in our department by adjusting the composition and proportion of herbs, and the development of the application of BTF in neurological disorders was based on the theory of the kidney-brain axis. It has been shown to effectively treat neurodegenerative diseases, including AD. Specifically, BTF has been proven efficient in practice in our clinical department for improving cognitive and memory functions in patients with AD. Our discoveries about the development of a chromatographic fingerprint for BTF through the modern analytical techniques, and characterization of the mechanisms by which BTF improved cognition provided a scientific basis for expanded use of this formula.

UPLC-MS combines the efficient separation capabilities of UPLC and the great power in the structural characterization of MS and provides a new powerful approach to identify the constituents in TCMFs rapidly and accurately [26]. In addition, diode array detection (DAD) is a commonly used detection technique for HPLC analysis. The combined use of DAD and MS can provide excellent specificity by providing orthogonal information for each peak. Fragmentation can be used to identify compounds using databases, and novel compounds can be identified using data deconvolution software sand spectral matching. In our current study, five components were identified that were associated with two herbs in BTF (Figure 1(b)). According to phytochemistry studies [27, 28], the main components of Gui-Ban and Long-Gu were amino acids with no conjugated bonds and minerals, respectively, which do not typically contain chromophores and cannot be detected by UV analysis. Moreover, the monitoring wavelength of 270 nm may not have captured the absorbance of asarone and saponins present in Shi-Chang-Pu and Yuan-Zhi, respectively. Therefore, UV detection may not be sufficient to characterize BTF [29, 30]. Furthermore, the lack of commercial standards for analysis and characterization may also be a limiting factor. In terms of the brain availability of the molecules we identified by UPLC-MS, those compounds belong to polyphenols (PPs). PP metabolites could indirectly regulate the cerebrovascular system or directly act as neurotransmitters crossing the blood-brain barrier, while the gut microbiota plays a crucial role in metabolizing dietary polyphenols into lipid-soluble metabolites that are absorbed by cells [31, 32]. In our experiment, we administrated BTF by oral gavage and believed that the metabolites of PPs that were transformed from the gut microbiota or live metabolism could pass through the blood-brain barrier (BBB) and accumulated in the brain.

A pathological hallmark of AD is the presence of amyloid plaques within the brain, an observation that led to the *β*-amyloid (A*β*) cascade hypothesis of AD [33, 34]. Attention has since focused on the isoforms and their physiological function, specially A*β*_1–42_. Recent studies have shown that AD may be caused by A*β* aggregates that adopt alternative conformations, resulting in prion-like self-propagation [35–37]. Intracerebroventricular injection of A*β*_1–42_ fibrils has been shown to induce hyperphosphorylation of tau, tangle formation, and leading eventually to neuronal death and dementia and has also been reported as seeds to induce endogenous A*β* aggregation then trigger neurotoxicity [33, 38]. These findings resulted in the development of models using endogenous A*β* and other plaque-associated factors without the need to overexpression of potentially confounding amyloid precursor protein (APP) domains, which naturally became a classic and popular AD animal model. Even so, further investigation on transgenic models of AD will be scheduled to fully assess the potential benefit of BTF and to exclude any side effect caused by impairment due to mechanical injury in the A*β*_1–42_ fibril-infused rats used in our studies.

BDNF exerts its biological functions on neurons through two transmembrane receptors: TrkB and p75 neurotrophin receptor (p75NTR) [39]. TrkB is a high-affinity catalytic receptor for several neurotrophins and is highly enriched in the hippocampus [40]. Phosphorylation of TrkB following stimulation by BDNF triggers the downstream activation of the PI3K/Akt/CREB signaling cascades, driving synaptotagmin and PSD95 synthesizing in endoplasmic reticulum (ER) and trafficking to synapses throughout the neurons, which results in rapid and dendrite-wide sensitization for synaptic potentiation [41, 42]. Intriguingly, BDNF gene transcription is controlled by the CREB family of transcription factors, and PSD95 interacts with TrkB receptor for BDNF [43, 44]. These two circulation pathways promote the high expression of BDNF and PSD95, as well as stable TrkB receptors for responding to BDNF stimulation effect. As a result, the activation of these pathways results in a positive feedback loop in which synapses become more responsive to BDNF, which leads to increased transport of PSD95 to synapses. Together, we noted that BTF significantly stimulated the expression of BDNF and subsequently induced the activation of the TrkB-Akt/CREB cascaded signaling pathways, which resulted in increased synthesis of downstream synaptotagmin and PSD95 in synapses. These results identified a potential mechanism of BTF-induced significant stabilization of synaptic plasticity in A*β*_1–42_ fibril-infused rats.

## 5. Conclusion

In conclusion, our study generated a chromatographic fingerprint for the Bushen-Tiansui Formula (BTF) that has been utilizing in our clinic department for several years and further confirmed that BTF improved cognitive function through the prevention of synaptic loss, as demonstrated in the classical AD model of A*β*_1–42_ fibril-infused rats. Furthermore, the mechanism investigation revealed that BTF stimulated the expression of BDNF and triggered the activation of the TrkB/Akt and TrkB/CREB pathways, which resulted in the upregulation of synaptotagmin and PSD95 expression in synapses. In short, this study provides a compelling scientific basis for expanding the application of BTF in the clinic, and the ongoing randomized clinical trial of BTF, in turn, will confirm the reliability of our reporting, which provides a reliable pathway for the treatment of AD.

## Data Availability

The data used to support the findings of this study are included within the article.

## References

[1] Takahashi R. H., Capetillo-Zarate E., Lin M. T., Milner T. A., Gouras G. K. (2010). Co-occurrence of Alzheimer's disease *β*-amyloid and tau pathologies at synapses. *Neurobiology of Aging*.

[2] Karran E., Mercken M., De Strooper B. (2011). The amyloid cascade hypothesis for Alzheimer's disease: an appraisal for the development of therapeutics. *Nature Reviews. Drug Discovery*.

[3] Scheff S., Price D., Schmitt F., DeKosky S., Mufson E. (2007). Synaptic alterations in CA1 in mild Alzheimer disease and mild cognitive impairment. *Neurology*.

[4] Liu Y. F., Chen H. I., Wu C. L. (2009). Differential effects of treadmill running and wheel running on spatial or aversive learning and memory: roles of amygdalar brain-derived neurotrophic factor and synaptotagmin I. *Journal of Physiology (London)*.

[5] Nakai T., Nagai T., Tanaka M. (2014). Girdin phosphorylation is crucial for synaptic plasticity and memory: a potential role in the interaction of BDNF/TrkB/Akt signaling with NMDA receptor. *The Journal of Neuroscience*.

[6] Wang S., Yu L., Yang H. (2016). Oridonin attenuates synaptic loss and cognitive deficits in an A*β*_1–42_-induced mouse model of Alzheimer’s disease. *PLoS One*.

[7] Zuccato C., Cattaneo E. (2009). Brain-derived neurotrophic factor in neurodegenerative diseases. *Nature Reviews Neurology*.

[8] Beck M., Flachenecker P., Magnus T. (2005). Autonomic dysfunction in ALS: a preliminary study on the effects of intrathecal BDNF. *Amyotrophic Lateral Sclerosis*.

[9] Ochs G., Penn R. D., York M. (2000). A phase I/II trial of recombinant methionyl human brain derived neurotrophic factor administered by intrathecal infusion to patients with amyotrophic lateral sclerosis. *Amyotrophic Lateral Sclerosis and Other Motor Neuron Disorders*.

[10] Lin Z., Gu J., Xiu J., Mi T., Dong J., Tiwari J. K. (2012). Traditional Chinese medicine for senile dementia. *Evidence-based Complementary and Alternative Medicine*.

[11] Tang Q., Ke H., Wu C. (2019). Aqueous extract from You-Gui-Yin ameliorates cognitive impairment of chronic renal failure mice through targeting hippocampal CaMKII*α*/CREB/BDNF and EPO/EPOR pathways. *Journal of Ethnopharmacology*.

[12] Zhang Y., Lin C., Zhang L. (2015). Cognitive improvement during treatment for mild Alzheimer’s disease with a Chinese herbal formula: a randomized controlled trial. *PLoS One*.

[13] Li L., Wei H., Zhang L., Chu J., Zhao L. (2006). Modern biological basis of Chinese medical theory that “kidney nourishes marrow and brain is sea of marrow”. *China Journal of Chinese Materia Medica*.

[14] Sheng C., Xu P., Zhou K., Deng D., Zhang C., Wang Z. (2017). Icariin attenuates synaptic and cognitive deficits in an A*β*_1–42_-induced rat model of Alzheimer’s disease. *BioMed Research International*.

[15] Goldsbury C. S., Wirtz S., Müller S. A. (2000). Studies on the in vitro assembly of A*β* 1–40: implications for the search for A*β* fibril formation inhibitors. *Journal of Structural Biology*.

[16] Luo H. B., Li Y., Liu Z. J. (2016). Protective effect of tetrahydroxy stilbene glucoside on learning and memory by regulating synaptic plasticity. *Neural Regeneration Research*.

[17] Fischer A. H., Jacobson K. A., Rose J., Zeller R. (2008). Hematoxylin and eosin staining of tissue and cell sections. *Cold Spring Harbor Protocols*.

[18] Yu J., Xie J., Mao X. J. (2012). Comparison of laxative and antioxidant activities of raw, processed and fermented Polygoni Multiflori Radix. *Chinese Journal of Natural Medicines*.

[19] Zhou D. A., Deng Y. N., Liu L., Li J. J. (2015). Effect of kidney-reinforcing and marrow-beneficial traditional Chinese medicine-intervened serum on the proliferation and osteogenic differentiation of bone marrow stromal cells. *Experimental and Therapeutic Medicine*.

[20] Shankar G. M., Walsh D. M. (2009). Alzheimer's disease: synaptic dysfunction and A*β*. *Molecular Neurodegeneration*.

[21] Kurella M., Mapes D. L., Port F. K., Chertow G. M. (2006). Correlates and outcomes of dementia among dialysis patients: the Dialysis Outcomes and Practice Patterns Study. *Nephrology Dialysis Transplantation*.

[22] Bugnicourt J.-M., Godefroy O., Chillon J.-M., Choukroun G., Massy Z. A. (2013). Cognitive disorders and dementia in CKD: the neglected kidney-brain axis. *Journal of the American Society of Nephrology*.

[23] Miwa K., Tanaka M., Okazaki S. (2014). Chronic kidney disease is associated with dementia independent of cerebral small-vessel disease. *Neurology*.

[24] Murray A. M. (2008). Cognitive impairment in the aging dialysis and chronic kidney disease populations: an occult burden. *Advances in Chronic Kidney Disease*.

[25] Wang Z. (2010). *The Essence of Qin-Jin Formulas*.

[26] Yang M., Sun J., Lu Z. (2009). Phytochemical analysis of traditional Chinese medicine using liquid chromatography coupled with mass spectrometry. *Journal of Chromatography A*.

[27] Wang C., Zeng H., Chen W. (2007). GC-MS fingerprint of effective components extracted from plastrum testudinis and its application. *Central South Pharmacy*.

[28] Zhang H., Zhang L., Liu Y. (2011). Studies on chemical components and pharmacological activities of Os Draconis (Longgu) and Ostreae Concha. *China Journal of Chinese Materia Medica*.

[29] Liu J., Yang X., He J., Xia M., Xu L., Yang S. (2007). Structure analysis of triterpene saponins in *Polygala tenuifolia* by electrospray ionization ion trap multiple-stage mass spectrometry. *Journal of Mass Spectrometry*.

[30] Shi H.-X., Yang J., Yang T. (2014). Alpha-asarone protects endothelial cells from injury by angiotensin II. *Evidence-based Complementary and Alternative Medicine*.

[31] Filosa S., di Meo F., Crispi S. (2018). Polyphenols-gut microbiota interplay and brain neuromodulation. *Neural Regeneration Research*.

[32] Schaffer S., Halliwell B. (2012). Do polyphenols enter the brain and does it matter? Some theoretical and practical considerations. *Genes & Nutrition*.

[33] Hardy J., Allsop D. (1991). Amyloid deposition as the central event in the aetiology of Alzheimer's disease. *Trends in Pharmacological Sciences*.

[34] Hardy J. A., Higgins G. A. (1992). Alzheimer's disease: the amyloid cascade hypothesis. *Science*.

[35] Goedert M. (2015). Alzheimer’s and Parkinson’s diseases: the prion concept in relation to assembled A*β*, tau, and *α*-synuclein. *Science*.

[36] Jucker M., Walker L. C. (2013). Self-propagation of pathogenic protein aggregates in neurodegenerative diseases. *Nature*.

[37] Watts J. C., Condello C., Stöhr J. (2014). Serial propagation of distinct strains of A*β* prions from Alzheimer’s disease patients. *Proceedings of the National Academy of Sciences of the United States of America*.

[38] Sowade R. F., Jahn T. R. (2017). Seed-induced acceleration of amyloid-*β* mediated neurotoxicity in vivo. *Nature Communications*.

[39] Kaplan D. R., Miller F. D. (2000). Neurotrophin signal transduction in the nervous system. *Current Opinion in Neurobiology*.

[40] Shelton D. L., Sutherland J., Gripp J. (1995). Human trks: molecular cloning, tissue distribution, and expression of extracellular domain immunoadhesins. *The Journal of Neuroscience*.

[41] McGinty J. F., Bache A. J., Coleman N. T., Sun W.-L. (2011). The role of BDNF/TrkB signaling in acute amphetamine-induced locomotor activity and opioid peptide gene expression in the rat dorsal striatum. *Frontiers in Systems Neuroscience*.

[42] Yoshii A., Constantine-Paton M. (2007). BDNF induces transport of PSD-95 to dendrites through PI3K-AKT signaling after NMDA receptor activation. *Nature Neuroscience*.

[43] Cao C., Rioult-Pedotti M. S., Migani P. (2013). Impairment of TrkB-PSD-95 signaling in Angelman syndrome. *PLoS Biology*.

[44] Tao X., Finkbeiner S., Arnold D. B., Shaywitz A. J., Greenberg M. E. (1998). Ca^2+^ influx regulates *BDNF* transcription by a CREB family transcription factor-dependent mechanism. *Neuron*.

